# Sugar labeling: How numerical information of sugar content influences healthiness and tastiness expectations

**DOI:** 10.1371/journal.pone.0223510

**Published:** 2019-11-04

**Authors:** Simona Haasova, Arnd Florack

**Affiliations:** Department of Psychology, University of Vienna, Vienna, Austria; University of Florida, UNITED STATES

## Abstract

Overconsumption of highly sugary foods contributes to increases in obesity and diabetes in our population, and initiatives are issued worldwide to reduce sugar content in food products. However, it is unclear how the presentation of reduced sugar content on food packages affects taste expectations of consumers. Based on the learned knowledge about negative health effects of sugar and the common belief that unhealthy food tastes better than healthy food, consumers might conclude that lower sugar levels are associated with higher healthiness and lower tastiness. Addressing this concern, we examined how quantitative information about sugar content without any verbal description influences consumers’ health and taste expectations of dairy desserts. We asked participants to indicate the expected healthiness and tastiness of randomly sampled dairy desserts, while varying systematically the quantitative sugar information provided in a label presented with the desserts (numerical sugar level in grams per 100 grams of product: low vs. original vs. high). We assumed that quantitative sugar content is not equally associated with healthiness and tastiness of products and that numerical information about sugar content informs health more than taste expectations. Therefore, we predicted that consumers expect higher healthiness, but not to the same degree lower tastiness for products with reduced sugar contented compared to products with higher sugar content. The results of the present study are in line with this hypothesis. We found that consumers expected desserts with less sugar to be healthier than desserts with higher levels of sugar. The experimentally varied sugar levels did not affect the tastiness expectations. Notably, consumers did not follow the unhealthy = tasty intuition and did not devaluate the tastiness of desserts because of heightened healthiness expectations. Our findings suggest that sole numerical information about sugar content—an important nutritional value—is more diagnostic in the construction of healthiness rather than tastiness expectations of food products.

## Introduction

In 2015, the EU High Level Group on Nutrition and Physical Activity (HLG) under the general European Union Framework for National Initiatives and Selected Nutrients initiated a program advancing reduction of sugar consumption among EU consumers. Identifying high added sugar in food products to be a contributing factor to diet-related health burden, the program aims to respond to the population growth in obesity [1] with monitoring and regulatory activities such as legal restriction of sugar content in food products, sugar-related taxes or salient presentation of sugar-content information on product packages. The EU member states jointly set the target for reduction of added sugar in food products sold to consumers in EU to the minimum of 10% by 2020 in comparison to the baseline in 2015 [2,3]. At about the same time, individual retailers launched initiatives to support mindful use of sugar. For example, in 2018, one of the leading supermarket chains started a nationwide campaign in Austria to sell dairy products with reduced sugar content [4]. Moreover, in other countries food producers responded to taxes on sugar sweetened beverages with reducing sugar content of some products and advertising sugar free varieties [5].

While the initiatives to reduce sugar content are desirable from the policy perspective, retailers and food producers might be worried that the decreased sugar content and its salient publication on product packaging might, as a side effect, lead to consumers’ expectation that the sugar reduced alternatives are less tasty than the original products. Indeed, research has found that the popular belief that healthy food is less tasty might lead to the effect that consumers avoid healthy products [6]. Taste remains one of the most important criterions for food choices [7, 8, 9], thus reduced taste expectations could negatively affect sales. However, a precondition for such negative side effects of reduced sugar content on taste expectations is that consumers really use the numerical information about the sugar content on product packages to form their expectations about both health and taste of the offered products. At present, it is not clear whether consumers rely on such sole numerical information to derive these kinds of expectations about the products. Previous research has mainly focused on the effects of explicit labels such as “reduced-fat” [10], “high in calcium” [11] or general “organic” labels [12] on health judgments. While explicit labels are clearly formulated and easy to understand, it is much more challenging to assess, for instance, how 30 g sugar affects the healthiness and tastiness of a product. Consumers have often problems to interpret numbers and understand such food information [13].

To address the concerns of retailers and consumers alike that reduced sugar content could negatively affect taste expectations, we examined how quantitative information about foods’ sugar content that is usually depicted on food packages influences consumers’ health and taste judgments of these food products. Specifically, we studied dairy products from the category “desserts” because this food category, along sugar sweetened beverages and breakfast cereals, is explicitly targeted by the EU sugar-reduction program [2,3]. It is important to stress that we did not intend to investigate the influence of health messages with an evaluative quality or persuasive character (i.e., health/nutrition claims or symbols referring to a particular health promotion system). Rather, our aim was to examine how the pure provision of the quantitative information about how much sugar is included in a particular product (e.g., 15 g of sugar in 100 g of product) should influence consumers’ health and taste expectations toward this product in situations preceding food choice and consumption.

We assumed that the sugar content presented as a numerical value in grams per 100 g of the product’s weight has an influence on both healthiness and tastiness expectations. But we also assume that the effect sugar content presented as a numerical value on tastiness expectations is much weaker in its effect size than is the effect on healthiness expectations. Our assumption was based on the reasoning that consumers have learned to directly link sugar to healthiness expectations, however that it is more difficult for them to translate a certain amount of sugar into taste expectations, which are usually dominated by familiarity (e.g., previous experiences with products) and sensory input (e.g., products’ package design).

## Theoretical background

Vast amount of research has shown that, at the point of food choice, the expectations towards foods are heavily driven by the way they are described by extrinsic cues, including the naming, labeling, communicating and design, as well as nutritional information [8]. Consumers use extrinsic cues, because the nutritional (healthiness) and sensory (tastiness) intrinsic properties of food are not possible to be experienced at this stage. Thus consumers form expectations about these properties through integration of a great variety of extrinsic cues [14, 15]. While we did not find studies about how numerical sugar content values affect healthiness and tastiness expectations of consumers, both subjective and scientific evidence suggests that consumers’ consideration of how much sugar a food product contains should influence the expectations of this products’ health.

First, experts, campaigns, and media reports have actively made consumers aware of the well-documented impact of excessive sugar consumption on obesity, diabetes and coronary heart diseases [16, 17]. Sugar has been long flag-marked by the modern medicine and public discourse as one of the direct determinants of foods healthiness and nutrition, and consumers are prompted to reduce its consumption for health improvements [18, 19]. Second, consumers attend to nutrition information when they want to make health judgments [20, 21, 22] or use it when it is salient on product packages [22]. Hence, there are hints that consumers use available quantitative information about the food’s nutritional content, whether provided directly on the product packaging or else, to construct their healthiness expectations. Indeed, at least for health-oriented consumers nutrition labeling helps consumers to identify foods healthiness correctly [23] and increases healthy food choices [24]. We therefore hypothesized that consumers in general expect products to be healthier when they contain less sugar, signaled by numerical information about its content (a number referring to the amount of sugar in grams per 100g of the unprepared product).

Foods health is what economists and marketers refer to as “credence” quality, which is difficult to ascertain even after consumption and its judgments rely fully on extrinsic (nutritional) and other intrinsic cues [9]. Meanwhile tastiness represents an “experience” quality that can be assessed after consumption [9]. While evidence shows that nutritional or content-related information can be also important for taste judgments (e.g., fat and calorie information) [25], many studies, which found a link between the content information and taste expectations, do not provide numerical information, but rather labels and flavor descriptions. Other researchers, for example, used the claim “now reduced in salt, better taste” [26]. Studies using numerical information are rare and inconsistent in results. Previous research used “reduced-fat spread 40% fat” or “full-fat margarine 80%” labels and found no effect of the variation of the label alone on sensory ratings of the product [10]. By contrast, it was reported that consumers perceived a bologna labeled as “light type Bologna, 10% fat” as less tasty than a “regular type Bologna, 20% fat” [27]. Others, on the other hand, found that a “low fat” label was associated with better taste, but only when numerical caloric information was simultaneously presented [25]. Importantly, these two studies [27, 25] used a description together with the numerical information and not solely numerical information. Indeed, verbal descriptions help consumers to activate cortical representations of a taste experience (for a review, see [28]). Even further, it seems that food descriptions of *sensory* (“rich and delicious”) rather than *factual* (“Monosodium glutamate”) nature are stronger at activating brain areas related to taste expectations [29]. Food’s tastiness is as a sensory experience mainly based on integration of information from various sensory sources such as actual taste, vision, smell, haptic or even auditory perception [30] and judgments of this experience rely on sensory stimulation [31]. A numerical value has to be translated into such a simulated taste experience, and research in other areas has shown that affect-laden information is more helpful for many individuals than numbers to form a mental image of a situation [32, 33]. Moreover, research showed that once sensory information is available, they take prime over other extrinsic cues (such as numerical content information) in formation of tastiness judgments [9, 34, 35]. Therefore, we suppose that numerical values of sugar content affect taste expectations to a much lesser extent then health expectations, because they provide little direct sensory description or flavor-related information. But we do not expect taste judgments not to be affected by sugar content at all. First, consumers might use non-numerical indicators for the sweetness of products on product packages that are related to sugar content, or base their judgments on previous taste experiences with the product or a similar one. Second, the consumers might use their health judgments that were based on the numerical information to derive speculations about the taste of a product. Research suggests that consumers can infer taste from healthiness of products in a reversed way [6]. The authors found that food with health labels compared to food without such labels led consumers to expect less tasty food. Because sugar is presumably closely related to health judgments, it is reasonable to assume that consumers infer products, which they perceive as healthier, to be also less tasty. However, recent research has shown that individuals vary in their belief that healthy food is not tasty and found even stable positive correlations between health and taste judgments [36, 37]. Therefore, we did not expect that the unhealthy tasty belief might compensate for the difficulty to directly use numerical information to derive taste expectations.

To sum up, we hypothesized that the numerical information will have a considerable effect on the judgment of healthiness of products, but will have a much smaller effect on taste judgments of the given products. We tested our propositions in a study in which we presented Austrian consumers with unknown dessert products, which were adapted from a foreign supermarket. To strengthen the ecological validity of the study, we used an individual random selection of products for each consumer, which was sampled from all products from that specific food category offered in the supermarket. Hence, the current study does not suffer from the limitation that only a small selection of products was used. We varied the sugar content that was presented as usual on product packages in Europe. Note that our study is only concerned with sugar content per se and not other sugar-related substances such as artificial sweeteners, high fructose corn syrup, stevia or honey. Participants were explicitly presented with information about the pure sugar content. We presented the products either with their original level of sugar content, with an increased level (+30%) or a decreased level (-30%), while we presented only the numerical value of sugar in grams per 100 g of the product’s weight, and not the percentage. Importantly, participants could not see the same product across different sugar levels. We tested the effects of the sugar level on taste and health expectations. Furthermore, we have also taken into account the attractiveness of product packaging to control for the effect of products’ visual pleasantness. Also, we considered possible moderators like the general health interest [7], food pleasure orientation [38], and the belief in the unhealthy tasty intuition [6] because these moderators have been shown to moderate the relationship between health and taste judgments in previous research [36]. In particular, it is reasonable to suppose that individuals with a high general health interest are more likely to rely on the sugar level in judgments than those with a low general health interest. Also, it is reasonable to assume that individuals with a high belief in the unhealthy tasty intuition show a stronger negative effect of a low sugar level on taste, because they perceive this sugar level as less healthy than the other sugar levels and rely on this healthiness in their taste judgment. Finally, we directly assessed the association between health and taste judgments to map whether the data supports the application of an unhealthy = tasty heuristic.

## Methods

### Participants

We used a representative sample of consumers from Austria (stratified by the demographic variables of age, gender, and educational background according to the Austrian population) recruited through an access panel (“Talk Online Panel”) who participated in the online study in exchange for a 2.50 € payment. A total of 256 consumers participated in the survey, of which 27 participants showed minimal variance in the healthiness and tastiness ratings, meaning that their responses had a standard deviation of 0, no individual correlations could be computed, and were therefore excluded. The final sample then consisted of 229 participants, from which 50.7% were women, with a mean age of 43.70 years (*SD* = 15.20) and a mean BMI of 25.49 (*SD* = 5.30). Participants’ educational backgrounds were diverse: 24.9% of participants had completed high school, 21.8% had completed secondary education, 17.5% had a degree from a vocational school or training institute, 12.2% had completed compulsory schooling, 23.1% had a university degree, and 0.4% had other degrees.

### Ethics statements

The present study obtained prior approval for human subjects research by an institutional review board of the Department of Applied Psychology: Work, Education, Economy, Faculty of Psychology, University of Vienna, and was conducted in accordance with the Declaration of Helsinki (revised 1983) and local guidelines of the Faculty of Psychology, University of Vienna. In each study, participants were informed about the aim of the study and the confidentiality of the data collection, and they gave their consent to participate. Participants could also withdraw at any time during the study.

### Design and procedure

We applied a multilevel within-subject design with three conditions (sugar level [reported in grams per 100g]: low vs. original vs. high) while we varied between-subject, which product was presented in which sugar level. The low sugar level was the original sugar content -30%. The high sugar level was the original sugar content +30%. We administered online questionnaires in which we presented participants with 15 actual products, which were randomly sampled for each participant out of all products offered by a foreign supermarket chain in the category “desserts” (n = 82) and asked them to judge the products on healthiness and tastiness. The products were always presented with their associated sugar level reported in grams per 100g. Participants evaluated five products of each sugar level category.

We instructed participants to rate their healthiness and tastiness expectations of these 15 products in two separate rating blocks, followed by a block where participants rated the attractiveness of the products appearance. To avoid potential biases due to effects of memory and reference-point on product evaluations, we randomized the order in which the healthiness and tastiness expectations were measured (healthiness or tastiness expectations first) and also the order of the presented products in each of the three rating blocks.

We asked participants to rate how tasty they estimated the presented products would be on a vertical 10-point rating scale, with response options ranging from 1 (*not at all tasty*) to 10 (*very tasty*). The healthiness expectations were assessed with a 10-point staple scale format: “How healthy do you estimate the presented product to be?” with response options ranging from +5 to +1 (indicating healthiness) and -1 to -5 (indicating unhealthiness), displayed vertically underneath each other without option labels. Participants were told, “+5 indicates very high estimate of a product’s healthiness,” and “-5 indicates very low estimate of a product’s healthiness.” For the sake of simplicity in the statistical analyses, the healthiness scores were subsequently recoded to reflect the same scale as the tastiness scores (*1 = very unhealthy* and *10 = very healthy*). By employing two different scales formats to measure the two constructs of healthiness and tastiness, we followed Podsakoff et al.’s [39] recommendations for procedural remedies to avoid the “common scale format” as a source of potential common method variance that due to correlational and proximate nature of our measurements could possibly inflate the found relationships between measured variables. After the assessment of tastiness and healthiness of the products, participants rated the attractiveness of the products appearance, answering the question “How attractive do you find the packaging design of the presented product?” with response options represented by a number of appraised stars ranging from 1 star (indicating unattractiveness) to 7 stars (indicating attractiveness). Afterwards, we asked about demographic variables (e.g., age and gender) and assessed individual characteristics: the explicitness of participants’ beliefs in the unhealthy = tasty intuition, a 2-item measure assessing the extent of the belief that unhealthy food tastes better than healthy food, example item: *“There is no way to make food healthier without sacrificing the taste”*, response options: 1 = *strongly disagree*, 9 = *strongly agree* [6]; general health interest, an 8-item measure assessing interest in eating healthily, example item: *“I always follow a healthy and balanced diet”*, response options: 1 = *strongly disagree*, 7 = *strongly agree*, [7] and food pleasure orientation, an 6-item measure assessing interest in eating healthily, example item: *“Enjoying food is one of the most important pleasures in my life”*, response options: 1 = *strongly disagree*, 7 = *strongly agree* [38]. Table 1 presents descriptive statistics for these variables and Cronbach’s alpha values for the scales (high values indicate high level of any of the given characteristics).

**Table 1:** Descriptive statistics and Cronbach’s alpha coefficients for the scales.

Variable	Study 1	
	*M* (*SD*)	α
Belief in unhealthy = tasty intuition	4.76 (2.08)	.76
General health interest	4.14 (1.00)	.76
Food pleasure orientation	5.00 (.97)	.69
Social desirability	1.28 (.17)	.69

### Materials

We used a representative sample of food products from the product category “desserts”, a subcategory of the product category “dairy” that were available for purchase in the online store of a Belgian supermarket, Delhaize, at the time of the study (August 2018). We chose rather unfamiliar products for the Austrian consumers to ensure our manipulation of products’ sugar content would not be compromised with consumers’ knowledge of the products original sugar contents. The 15 presented products were first randomly sampled for each participant from a pool of 82 items (e.g., mousses, puddings, rice puddings) and then randomly paired with one of the three sugar levels (low, original, high) ascribed to that particular product, so that each participant would judge five products from each sugar level condition. A random selection from a bigger pool of products was applied to increase the generalizability of the results. A shortcoming of experimental research is often that products are preselected that might best support the hypotheses of the researchers [40, 41]. With the currently applied design, we avoided this criticism, because we did not preselect single products from the product category. Moreover, we avoided showing one product with different sugar levels to participants to reduce demand effects and to create a situation, which is similar to real product exposures in online supermarkets. Products were presented as product pictures with a separate label clearly stating the products’ sugar content in grams of sugar per 100 grams of the product–the original sugar content was taken from the products official nutrition information, while lower sugar level was calculated as 30% less than original sugar content and high sugar level was calculated as 30% more than original sugar content of each particular product (for an example of the experimental material, please see the S1 Fig). We chose +/- 30 percent sugar levels in addition to the original level, because the 30 percent reduction level is relevant for the food industry. According to EU regulations [42] a claim stating that the sugar content has been reduced, may only be made where the reduction in content is at least 30% compared to a similar product.

### Data analysis

In our study, participants repeatedly evaluated multiple food products with varying characteristics. Thus, the evaluations were nested within participants and were intercorrelated. We therefore accounted for this issue by using a linear mixed effects model analysis [43, 44] as a more stringent test of our hypotheses alongside a correlation analysis. A significance level of α = .05 was adopted for all of the following analyses. All analyses were performed with SPSS and R [45], specifically the packages lme4 [46] and nlme [47]. The data supporting this study is freely available in a public repository. To access the data (and associated analyses), see the Research Data.

## Results

To test our hypothesis that the manipulated sugar levels of real food products will influence the healthiness judgments to a larger extent than the tastiness judgments, we used the basic linear mixed-effect model. In the basic model (Model 1, in Table 2), product evaluations were specified as the dependent variable, with sugar level (low, original and high) and evaluation type (healthiness, tastiness) and importantly, their interaction as predictors. The dependent variable of product evaluations encompassed both healthiness and tastiness evaluations of the given products, in order to be able to test whether the sugar-level conditions affected the type of evaluation differently.

**Table 2:** Parameter estimates of the effects of sugar level and evaluation type on products evaluations, using LMM.

Parameter	Model 1(basic)	Model 2	Model 3	Model 4
*Fixed effects*				
Intercept	4.09*** (.11)	4.09*** (.11)	4.09*** (.10)	4.00*** (.13)
Sugar level	-.65*** (.04)	-.65*** (.04)	-.66*** (.04)	-.66*** (.04)
Evaluation type	2.21*** (.13)	2.21*** (.13)	2.21*** (.13)	2.21*** (.13)
Sugar level x Evaluation type	.68*** (.05)	.68*** (.05)	.68*** (.05)	.68*** (.05)
General health interest		.01 (.09)	-.00 (.08)	-.01 (.08)
Belief in the unhealthy = tasty intuition		.17*** (.04)	.15*** (.04)	.15*** (.04)
Pleasure orientation		.19* (.09)	.19* (.08)	.19* (.08)
Product attractiveness			.25*** (.01)	.25*** (.01)
Order of evaluations				.17 (.16)

*Note*. Values are parameter estimates predicting the evaluations of products. Standard errors appear in parentheses. The continuous variables in the model are centered on grand mean. Sugar level is a dichotomous variable coded as follows: -1 = “low sugar,” 0 = “original sugar”; 1 = “high sugar”.

+p < .10.

*p < .05.

**p < .01.

***p < .001.

Evaluation type is a dichotomous variable coded as follows: 0 = healthiness evaluation, 1 = tastiness evaluation. Order of evaluations is a dichotomous variable coded as follows: 0 = healthiness preceding tastiness evaluations; 1 = tastiness preceding healthiness evaluations.

In the subsequent models (Model 2, 3, 4 in Table 2), we also controlled for participants individual characteristics (general health orientation, belief in the unhealthy = tasty intuition and pleasure orientation), products attractiveness and order of evaluation types. We included a random intercept per participant to account for by-subject variation. We also included random slopes for the effects of the evaluation type and sugar level to account for by-case variability in the effects of evaluation type and sugar level. We centered continuous predictors on their grand mean (an average of the participants means). The basic model was established as the result of systematic model fit testing in which we compared models that included random effects with models that excluded them. Parameters were estimated with a maximum likelihood estimator.

Most importantly for our hypotheses, the analyses (Table 2) showed a significant interaction between products’ sugar level and type of evaluation, suggesting that products’ sugar level was differently associated with healthiness and tastiness evaluations. Moreover, a significant main effect of the manipulated level of sugar on the general product evaluations indicated that products with lower sugar level had overall the highest product evaluations (*M*_*low*_ = 5.53, *SD*_*low*_ = 2.41; *M*_*original*_ = 5.15, *SD*_*original*_ = 2.67; *M*_*high*_ = 4.89, *SD*_*high*_ = 2.67). The main effect of the evaluation type indicated that evaluations of products tastiness (*M* = 6.29, *SD* = 2.43) were in general higher than evaluations of healthiness (*M* = 4.09, *SD* = 2.34). The observed effects of the sugar level, evaluation type and their interaction predicting products evaluations did not change in significance after we controlled for product attractiveness and order of the healthiness and tastiness assessment in the model (Model 3 and 4, Table 2).

Because our manipulation of sugar levels consisted of numerical rather than verbal information, we have additionally also controlled for participant’s education level in every step of the analyses in Table 2 to make sure that participants’ education and ability to translate numerical information into meaningful interpretations did not play a role in our results. The results remained unchanged after doing so.

**Fig 1:**
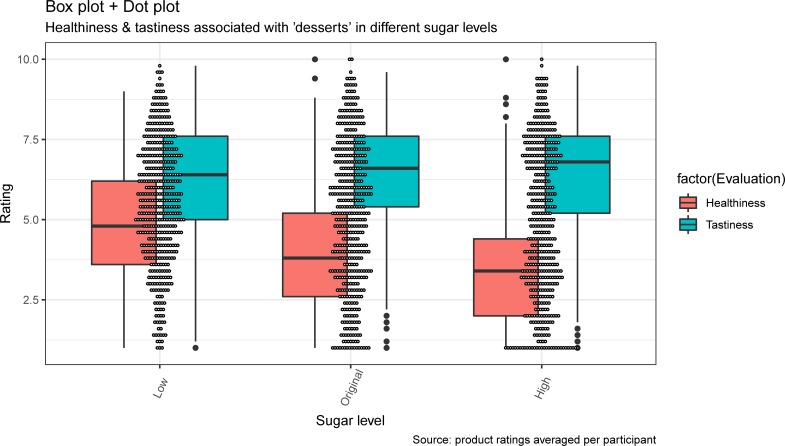
Box plot and dot plot depicting the healthiness and tastiness ratings of products with low, original and high sugar level content. *Note*. The top of the box represents the 75th percentile and bottom of box is 25th percentile, while the dark line inside the box is the median. The end points of the lines (whiskers) are at a distance of 1.5*Inter Quartile Range (the distance between 25th and 75th percentiles). The dots show the distribution of participants’ average healthiness/tastiness product ratings across products with different sugar levels.

To handle the interaction, we have further performed a repeated measure ANOVA analyses with a multilevel model, separately for the healthiness and the tastiness product evaluations. This basic model specified healthiness (tastiness) evaluations as dependent variables and sugar level as an independent variable, while accounting for participants random intercept and the within subject nature of the sugar-level condition (random slope). The planned contrasts (for means and standard deviations of healthiness and tastiness evaluations between the sugar level conditions, see Table 3) revealed that consumers evaluated products with low sugar content as healthier than products with original sugar content (*b* = .90, *t*(456) = 11.29, *p* < .001) and products with original sugar content as still healthier than products with high sugar content (*b* = .41, *t*(456) = 5.10, *p* < .001). The results remained unchanged after controlling for products tastiness evaluations, individual characteristics, products attractiveness and order of evaluations. The sugar level did not seem to affect the tastiness evaluations in similar or another way as tastiness of products lower in sugar was evaluated similarly as with their original sugar content (*b* = -.14, *t*(456) = -1.74, *p* = .082), which were evaluated similarly to products higher in sugar content (*b* = .10, *t*(456) = 1.22, *p* = .222). Visualization of the differentiating effects of sugar level on tastiness and healthiness product evaluations can be seen in Fig 1. The results remained unchanged after controlling for products healthiness evaluations, individual characteristics, products attractiveness and order of evaluations.

**Table 3:** Mean healthiness and tastiness ratings of products with low, original and high sugar level contents.

	Healthiness	Tastiness
	*M* (*SD*)	*M* (*SD*)
Low sugar	4.82 (2.29) _a_	6.23 (2.32) _a_
Original sugar	3.92 (2.31) _b_	6.38 (2.43) _a_
High sugar	3.51 (2.22) _c_	6.27 (2.52) _a_

*Note*. Means with different subscripts between rows are significantly different at p < .001 in paired contrasts.

These findings indicate that healthiness expectations, unlike the tastiness expectations, were negatively associated with the manipulated increase of sugar content.

### Moderation analyses

In light of our hypotheses and results indicating that numerous variables representing consumers’ individual attitudes were related to products healthiness evaluations, we have performed multiple moderation analyses, using the previously specified basic model for separate analyses of healthiness evaluations. To the basic model, we added an interaction term between the sugar-level condition and the moderating variables (general health interest, belief in the unhealthy = tasty intuition, and food pleasure orientation). The statistical assessment of these interaction effects on healthiness judgments revealed no significant effects, thus no moderation of the effect of sugar level by individual characteristics on healthiness could be shown.

### Association between health and taste expectations

Considering all single-product judgments, we observed a significant positive correlation between healthiness and tastiness expectations: *r*(3435) = .190, *p* < .001 (95% CI [.157 - .222]; aggregated across single judgments: *r*(229) = .348, *p* < .001).

Also, we calculated correlations between healthiness and tastiness expectations on the individual level. We found that the relationship varied from positive to negative and weaker to strong in each of the (low, original, high) sugar-level conditions as indicated by the interquartile range and the median value of the healthiness-tastiness correlation coefficients per participant in each of the sugar-level conditions (Tables 4 and 5).

**Table 4:** Range and median values for the general correlation between healthiness and tastiness ratings.

	General	
Healthiness-tastiness correlation		
	Interquartile range* (*r*)	Median (*r*)
Overall (N = 229)	-.29 –.28	-.02

**Table 5:** Range and median values for correlations between healthiness and tastiness ratings across sugar-level conditions.

	Sugar-level condition: low sugar			original sugar			high sugar	
	Healthiness-tastiness correlation							
	Interquartile range* (*r*)	Median (*r*)		Interquartile range* (*r*)	Median (*r*)		Interquartile range* (*r*)	Median (*r*)
Overall (N = 221)	-.44 –.38	-.08	Overall (N = 207)	-.49 –.40	-.08	Overall (N = 193)	-.46 –.41	-.07

* Interquartile range = 25^th^– 75^th^ percentile.

However, when using a LMM accounting for the within-subject nature of our data (including the random intercept and random slope of the predictor) to test the relationship between healthiness on tastiness expectations, we found no significant effects of healthiness judgments predicting tastiness judgments (*b* = .05, *t*(3205) = 1.474, *p* = .141) and vice versa (*b* = .02, *t*(3205) = 0.792, *p* = .428).

## Discussion

To stop dangerous population trends in obesity, organizations like the World Health Organization [48] are calling for a global reduction of the amount of sugar in food products. While many global players in food industry have launched product lines promoting healthier consumption by reducing sugar content in their own products, their campaigns often emphasize the maintenance of the “original” product`s taste despite the sugar reduction, e.g., Coca-Cola [49] and Nestlé [50], signaling the worry that consumers’ might expect the sugar reduced alternatives to be less tasty than the original products. To clarify whether and to what extent numeric sugar content of food products influences healthiness and tastiness expectations, the present study presented consumers with various dairy desserts and their sugar content, where the ostensible numeric amount of sugar per 100 gram of the product was either the original sugar value or value experimentally increased (+30%) or decreased (- 30%). We hypothesized that the level of sugar content (low, original, high) affects healthiness expectations substantially more than tastiness expectations, because consumers are more readily connecting increasing level of sugar with lower health. By contrast, we assumed that expectations of tastiness are more strongly driven by previous experiences and sensory information. Our data showed that the provided numeric information about the level of sugar content is well reflected in healthiness expectations but not in tastiness expectations. Consumers judged dairy desserts with least sugar as the healthiest ones, while their judgment of tastiness did not change with the differing numbers of sugar content of the desserts. The current study assessed consumers’ expectations of healthiness and tastiness. These expectations represent important determinants of actual product choice and purchase intentions of unfamiliar products [51, 52, 6]. The current findings therefore directly apply to those frequent situations when a consumer cannot remember the concrete taste of a given product or when she did not taste the product before, as it is the case for novel products or for existing products consumers did not try yet, or consumes less frequently.

Previous research has shown a similar pattern of results in regard to the differential influence of nutrition-related information on health and taste judgments. For example, Lee, Shimizu, Kniffin and Wansink [53] found that food labeled as organic was consistently rated as healthier (less caloric, more nutritious). However, no consistent results on taste judgments of products were apparent in these studies. Further, Wang, Oostindjer, Amdam, and Egelandsdal [54] found in a related study that nutrition labels had an effect on health, but not on taste judgments. One explanation for these differential effects of numeric information on health and taste judgments is that consumers have learned the meaning of numeric nutrition information from various sources, like the media and parental and school education and apply this knowledge to form expectations about foods´ healthiness.

Usually, such salient nutritional information is utilized in the form of simple rules of thumb [38, 55] for instance, when consumers think that healthier means less calories [56]. In accordance, our results suggest that in a manner of a simple rule of thumb, consumers were able to easily use the provided value of products sugar content to determine products healthiness such that higher sugar signified lower healthiness and vice versa.

The present results do not support the reservation that consumers do not know how to effectively translate a quantitative nutrition value into the concept of healthiness or tastiness without a particular informative context or provided anchors. Our data suggest that consumers possess a basic understanding of the relationship between sugar content and healthiness and have some individual anchors of what represents little to lot of sugar. Indeed, sugar is one of the most frequently used nutrition-related information and also other studies are in line with the idea that consumers can use sugar-related information for simple assessments [57].

Because the present study is based on survey data and did not observe actual behavior, we cannot rule out that consumers’ health ratings are a consequence of socially desirable answering and impression management [58]. The participants might have attempted to show their knowledge about healthy eating and to appear as “healthy eaters”. Without the observation of purchase behavior, we cannot conclude that the health judgments have any positive effect of purchasing the less sugared products or indeed prevent the avoidance of this food. However, the survey was anonymous and participants had no reason to hide their real preferences. Also, participants did not evaluate the less sugared desserts better than the desserts with increased amounts of sugar, which might have reflected socially desirable answering strategies. It is also important to note that this study presented consumers with products of unfamiliar brands. While consumers might often seek variety and purchase products they have not purchased before, they also possess specific preferences for products and brands they purchase frequently. For such products consumers are likely to have concrete taste experiences already. The present study does not allow definite conclusions about how consumers would respond to a reduction of the sugar content of such products. We suppose that consumers show the same neglect of the numerical sugar information when forming their taste expectations for such products, while they use the information for their health judgments. Cheung and colleagues [59] have found that consumers are often blind to changes of ingredient information, but are more likely to notice them when they assess the naturalness of products, which is an aspect related to the healthiness of products. Future research might be dedicated to testing these propositions in a context, when well-established products change their sugar-content because of changing brand policies or new tax rules.

Taken together, the findings of the present study largely support our hypothesis that sole numerical information of an important nutritional value–sugar–is more diagnostic in the construction of health rather than taste perception of food products. It seems that labels conveying a lower quantitative amount of sugar in grams per 100 grams of the given product are not associated with a lower taste quality and therefore do not generate lower taste expectations. We suppose that in comparison to healthiness, tastiness represents a qualitatively different aspect of food and that healthiness and tastiness expectations are constructed partly differently. For once, food’s health is an abstract cognitive construction, while tastiness represents an experience quality [30, 9]. Nevertheless and as we argued earlier, using verbal descriptive labels rather than sole numerical information might be more relevant for taste expectations as indicated by research of Bialkova, Sasse, and Fenko [60] who found the label “30% less sugar” to decrease taste perception of cereal bars.

It is reasonable to assume that sole numeric information does not help to simulate the taste experience to form an expectation about the tastiness, but that it can be used easily for a health judgment, which is based on syllogistic reasoning.

Interestingly, the effect of sugar content level on healthiness judgments was not moderated by any measured health or taste related individual attitudes (general health interest, food pleasure orientation, belief in the unhealthy = tasty intuition). We speculate the reason for this absent finding might be the explicitness with which numerical sugar content is connected with food’s health aspect and the consequent easiness with which one can understand and apply this information. Forming and using the simple rule of thumb that higher sugar equals less healthiness might be universally easy, regardless of individually pronounced eating-related attitudes.

In addition, our findings are not consistent with the unhealthy = tasty intuition [6], which posits that consumers knowledge that a food is healthy unfavorably colors their taste expectations. Participants did not expect more sugary food (which was rated as less healthy) to taste better. Also, our data does not suggest that indication of the sugar content information on food product packaging leads to experience of reactance in consumers. This might present an advantage of this kind of strategy for health promotion, as verbal and persuasive health messages often do trigger reactance and can backfire such that consumers rather engage in the opposite of the recommended behaviors as an act of freedom restoration [61, 62, 63, 64]. Follow-up studies could employ taste tests of products with and without the quantitative labels to study the dynamic interplay between expectations and taste experiences. A further interesting point would be to study whether consumers make assumptions about other product ingredients depending on the level of sugar content and whether this is also the case when the sugar content is given solely in numerical form and when no verbal labels (e.g., reduced sugar) are provided.

To sum up, we showed in the present research that consumers’ shape their expectations about food products’ healthiness, but not tastiness, according to information on products’ sugar content presented in a numerical form. The lower the sugar content per 100 g of any given food product, the healthier consumers perceived the product to be. Importantly, the decrease of numerical sugar content did not lead consumers to the expectation that the food is less tasty. Consumers did not follow the unhealthy = tasty intuition when using the sugar content information for their healthiness (and tastiness) judgments. In the present study, we selected desserts as a product category (mostly consisting of dairy products) because it is among those categories that are targeted by the EU program on sugar reduction. Hence, we chose a highly relevant category for sugar reduction. Further research needs to test whether the effects of numerical information about the sugar content are different for other product categories. It is conceivable that the positive effect of reduced sugar on healthiness expectations is less pronounced and the effect on tastiness is different for products in categories, which are not typically associated with sweetness, such as starchy foods or soups.

Our data implies that the simple provision of quantitative nutritional information helps consumers to differentiate between healthier and less healthy food options. In this way, the numerical sugar information could help consumers to follow the recommendation of nutrition advisors to keep their daily intake of free sugars to less than 10% (approximately 6 sugar cubes or 60 g of sugar) of their total energy intake necessary for reduction of health risks [45]. The notion that such strategy does not necessarily alter consumers taste expectations of the food product poses a benefit for success of both global health campaigns as well as brands’ marketing.

## Supporting information

S1 Fig Experimental material. Example of experimental material presented to participants in our study–consisting of simultaneous presentation of a product and its associated sugar content. Please note that this picture serves an illustrative purpose and due to copyright reasons, here we used only a graphical icon to indicate the presented food products.
(PDF)
