# Supplementary material for: Sugar labeling: How numerical information of sugar content influences healthiness and tastiness expectations
Source: PLoS One. 2019 Nov 4;14(11):e0223510. doi: 10.1371/journal.pone.0223510 (PMC6827896; doi:10.1371/journal.pone.0223510)

S1 Fig. Experimental material. Example of experimental material presented to participants in our study – consisting of simultaneous presentation of a product and its associated sugar content. Please note that this picture serves an illustrative purpose and due to copyright reasons, here we used only a graphical icon to indicate the presented food products.

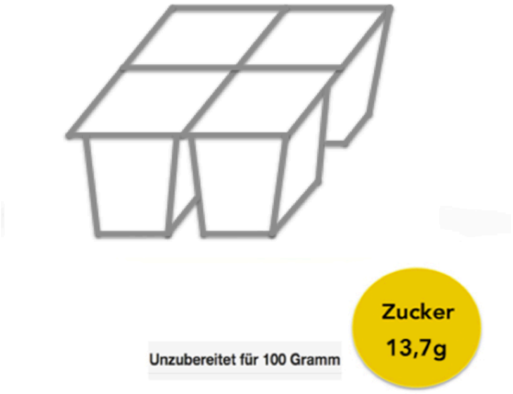

Supplement: S1 Fig — Example of experimental material presented to participants in our study–consisting of simultaneous presentation of a product and its associated sugar content. Please note that this picture serves an illustrative purpose and due to copyright reasons, here we used only a graphical icon to indicate the presented food products. (PDF) [file pone.0223510.s001.pdf]
